# Underlying IPEX syndrome in a patient with idiopathic juvenile arthritis and vitiligo

**DOI:** 10.1186/s13223-022-00740-9

**Published:** 2022-12-12

**Authors:** Leonardo Oliveira Mendonça, Adriana Pitchon dos Reis Chuster, Mayra Barros Dorna, Samar Freschi Barros, Janaina Baptista Alves, Victor Lucas Gonçalves, Ariana Campos Yang, Jorge Kalil, Myrthes Anna Maragna Toledo-Barros, Cristina Maria Kokron

**Affiliations:** 1 Division of Clinical Immunology and Allergy, Hospital das Clinicas of the Faculdade de Medicina of the Universidade de São Paulo, Department of Internal Medicine, Universidade de São Paulo, Rua Dr. Enéas de Carvalho Aguiar, 255, 8th floor, São Paulo, 05403-000 Brazil; 2 Laboratory for Immunological Investigation (LIM-19), Heart Institute, University of São Paulo, São Paulo, Brazil; 3 Division of Clinical Immunology and Allergy, Center for Rare and Immunological Disorders, DASA - Hospital 9 de Julho, São Paulo, Brazil; 4 Division of Allergy and Immunology, Department of Pediatrics, Faculdade de Medicina da Universidade de São Paulo, São Paulo, Brazil; 5 Department of Surgical Pathology, School of Medicine, University of São Paulo, São Paulo, Brazil

## Abstract

**Background:**

IPEX syndrome is an X-linked inborn error of immunity clinically characterized by the triad of: enteropathy, polyendocrinopathy and eczema. However many other clinical presentations lacking the triad above described have been reported what underpin the need of careful clinical suspicion, immunological evaluation and genetic sequencing.

**Case presentation:**

Here we report a case of a Brazilian boy with severe eczema as the first and only presentation requiring cyclosporin therapy. Progressive and cumulative symptoms of arthritis and enteropathy lead to the suspicion of an inborn error of immunity. Peripheral FOXP3 expression was normal (CD127−/CD4+/CD25+/FOXP3+—396 cells—63%) and a pathogenic mutation in FOXP3 gene (c.1150G>A; p.Ala384Thr), confirmed the diagnosis of IPEX syndrome.

**Conclusions:**

IPEX syndrome should be suspected in patients presenting with severe eczema associated or not with other autoimmune/hyper inflammatory diseases in life. Our study also reinforces that FOXP3 expression by flowcytometry seems not to be a good screening method, and genetic sequencing is mandatory even in those with high suspicion and normal peripheral FOXP3 expression.

**Supplementary Information:**

The online version contains supplementary material available at 10.1186/s13223-022-00740-9.

## Introduction

IPEX syndrome (MIM #304790) also known as immune dysregulation, polyendocrinopathy, enteropathy, X-linked is a monogenic inborn error of immunity due to loss-of-function mutations in the forkhead box 3 (FOXP3) gene. This gene is crucial for the development, maturation and maintenance of CD4^+^ regulatory T (T-reg) cells. Diverse phenomena mainly of autoimmune origin are characteristic of this syndrome such as enteropathy, endocrinopathies, cytopenias, renal disorders and skin manifestations [1].

IPEX syndrome presentation occurs very early in life, usually in the first month, and the median time to diagnosis vary from 1.16 to 2.5 years. As expected for an X-linked disorder, only male subjects are affected. Enteropathy and eczema is a common feature for very early (< 1 month) and early (> 1 month) presentation, whereas endocrinopathies, specially Type 1 diabetes seems to be more frequent only in those with very early presentation. Additional features, such as hematological and recurrent infections seems to be more frequent in the disease presentation after the first year of life. Immune mediated phenomena such as arthralgia or transient arthritis seems to be rare in IPEX as it affects 5.1% (2/39 patients) of the already reported FOXP3-IPEX patients. Furthermore, skin involvement in those patients is mainly characterized by eczema or dermatitis in both, single and multiple organ involvement [2].

Here we report the case of a Brazilian patient with juvenile idiopathic arthritis, eczema and vitiligo as the clue for the late diagnosis of IPEX syndrome harboring the A384T mutation in the FOXP3 gene.

## Patients and methods

### Clinical data and genomic sequencing

We retrieved clinical data from patient’s records, after parents signed a written consent for the publication of any potentially identifiable images or data included in this article. Genomic DNA was extracted from blood samples using a QIAamp^®^ DNA Blood Maxi Kit (Qiagen^®^, Valencia, CA, USA) and Peripheral blood mononuclear cells (PBMC) were obtained by density gradient centrifugation (*d* = 1.077 g/mL). We designed direct primers to FOXP3 gene exon 12 and performed Sanger sequencing for genetic confirmation and familial segregation using standard procedures.

### Quantification of lymphocytic FOXP3 phenotypes by flow cytometry

Patient’s PBMC previously separated by Ficoll density gradient centrifugation and cryopreserved were unfrozen, washed with 1 × PBS and stained with titrated mouse anti-human monoclonal antibodies (mAbs). Regulatory T cell subset was characterized by: anti-CD3 FITC, anti- CD4-AmCyan, anti-CD8 Pacific Blue, anti-CD127 PE, FOXP3 PerCp-Cy5.5, CD28 APC and CD25 PE-Cy7. All mAbs were from BD Biosciences, except Foxp3 (eBioscience/Invitrogen). A viability marker was added: FVS 780 (BD Biosciences). Surface staining (mAbs diluted in cold 1× PBS) was performed for 30 min at 4 °C. For intracellular staining of FOXP3, cells were washed, fixed and permeabilized with Transcription Factor Buffer Set (BD Biosciences) immediately after surface staining, according to the manufacturer's instructions. Fluorescence minus one (FMO) controls were set up for FOXP3 markers.

## Results

### Case report

The patient is a 15 years old boy born from non-consanguineous parents from the state of São Paulo, Brazil. He was sent to our evaluation at the age of 4 years due to a recalcitrant eczema at that time associated with a possible milk and egg allergy. In addition, the patient had rhinitis and asthma with a negative skin prick test for the most common inhalant allergens in Brazil including house dust, fungi, animal epithelium and cockroach. An oral food challenge with egg and milk came out negative and was done to better understand if any of these agents was a possible trigger of the eczema. The first skin biopsy found acanthosis, parakeratosis and agranulosis in the epidermis with micro abscess in the stratum corneum. In the papillary dermis was found vascular ectasia with a mix of inflammatory cells with neutrophilic predominance, both signs consistent of chronic eczema. Topical treatment with corticosteroids and tacrolimus was not effective in controlling the eczema. Due to the recalcitrant skin condition, the patient was medicated with oral cyclosporine (dose 2.5–4.2 mg/kg/day) for three years and discontinued after amelioration.

At the age of 7 years during routine laboratory work up a selective IgA deficiency (IgA levels of 0.4 mg/dL) was found. Also at seven, the patient started begun to present diffuse arthralgia with enlargement of knees and the diagnosis of Idiopathic Juvenile Arthritis was made. Another round of immunosuppression with Anti-TNF (Etanercept—dose 45 mg/2 weeks) and methotrexate (17.5 mg/week) achieved clinical control of articular conditions and no relapse of skin condition was observed for five years. At the age of 12 years, while still on MTX and anti-TNF therapy he had onset of new symptoms that included alopecia areata, vitiligo, and diarrhea without blood or mucus.. A colonoscopy was performed and lymphangiectasis and diffuse colitis was found which led to a presumed diagnosis of inflammatory bowel disease. Anti-TNF and methotrexate were discontinued and oral mesalazine was initiated with partial resolution of the diarrhea and no worsening of the arthritis. Because of the initial suspicion of IPEX syndrome, the patient was screened for autoimmune diseases including endocrinopathy and anti-gad, ANA-antibodies and rheumatoid factor was negative. No growth alteration was observed over those years.

**Fig. 1:**
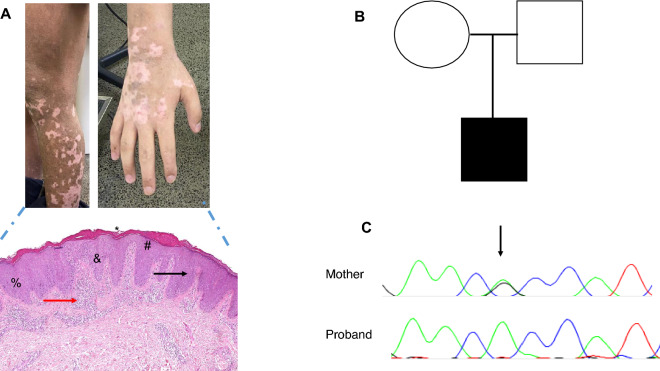
Skin and pathological findings (**A**), familial pedigree (**B**) and electropherograms (**C**). **A** Vitiligo-like lesions observed in the legs and hand. Skin biopsy (left forearm), histopathological findings are: the epidermis shows parakeratosis, areas of hypogranulosis, acanthosis and mild spongiosis. The dermis exhibits a perivascular and superficial inflammatory infiltrate of lymphocytes and scattered neutrophils, as well as fibroplasia and verticalized papillary collagen fibers (hematoxylin–eosin, ×100). **B** Familial pedigree evidencing the affected proband in black quarter. **C** Electropherograms evidencing the c.1150G>A; p.Ala38Thr mutation in FOXP3 found in the affected proband and in the mother

**Fig. 2:**
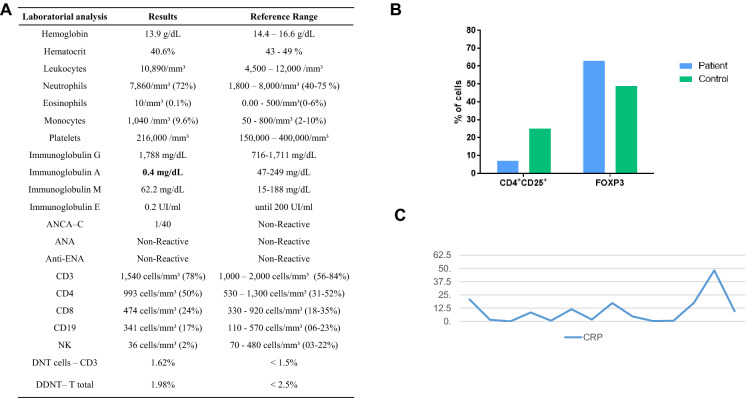
Baseline laboratory findings and peripheral FOXP3 expression. In **A**—Baseline laboratory findings and in bold absent levels of IgA. In **B**—Quantitative expression of FOXP3 (CD127–/CD4+/CD25+/FOXP3+) in the proband and in a healthy control age and gender matched. **C**—Fluctuating levels of CRP (C-reactive protein—reference range < 5 mg/dL) observed over the years

Because of the suspicion of IPEX, peripheral quantification of FOXP3 was performed at the age of 14, when the patient was under oral mesalazine and two years free of anti-TNF and methotrexate, which resulted normal. Nevertheless, with the high suspicion of IPEX a genetic sequencing was requested that came out positive to an already reported mutation in the FOXP3 gene. After genetic diagnosis, the patient was sent to bone marrow transplant. All relevant laboratory analysis, clinical and pathological results are shown in Figs. 1 and 2.

### Genetic analysis

Due to a suspicion of an inborn error of immunity a target gene panel of 207 genes (Invitae Primary Immunodeficiency Panel) was requested. An already reported mutation in the FOXP3 gene was found c.1150G>A; p.Ala384Thr, exon 12. The mutation was therefore confirmed in the index patient and in his mother by Sanger sequencing (Fig. 1).

### Peripheral quantification of FOXP3 lymphocytes

Normal levels of CD3 (1540 cells—78%); CD4 T cells (993 cells—50%) and high FOXP3 (CD127−/CD4+/CD25+/FOXP3+) expression (396 cells—63%) in the proband when compared to a healthy control (CD127−/CD4+/CD25+/FOXP3+—823 cells—48.9%) (Fig. 2). The patient had not been on anti-TNF and methotrexate for two years and was on oral mesalazine for two years at the time these investigations were completed. All FMO files and plotting figures of patient, his mother and healthy controls are in Additional file 1.

## Discussion

Here we report the case of a late diagnosis of IPEX syndrome in a Brazilian patient harboring an already reported mutation in FOXP3 gene with a mild phenotype.

Clinical phenotype linked to IPEX syndrome were originally attributed to the acronym of IPEX (immune dysregulation, polyendocrinopathy and enteropathy) inherited in a X-linked pattern [1]. However, many cases have already been reported lacking the classical phenotype and with late onset, as here reported [3–8]. Special attention should be paid to patients like herein reported that initially present with a single symptom, such as eczema, and that progressively accumulate additional findings leading to delay in diagnosis of an Inborn Error of Immunity [9, 10]. Of note, it is of great relevance that the clinical course herein described may have been impacted due to the use of immunosuppressive treatments over the years and this also impacted the early recognition of IPEX syndrome. On the other hand, benefit of the use of both, cyclosporine, methotrexate and anti-TNF has been reported in non-transplanted IPEX patients [11].

With this report we also expand the clinical findings of IPEX syndrome. Skin manifestations is a common finding in IPEX but vitiligo has not yet been reported although FOXP3 gene seems to be relevant in individuals affected by vitiligo [12]. Additionally, immunedysregulatory findings consistent with juvenile idiopathic arthritis is also uncommon description in IPEX syndrome [2]. Such observations are very important not just to expand the constellation of symptoms of IPEX syndrome but also to highlight the necessity to consider genetic screening in patients harboring multiple autoimmune/inflammatory phenomena.

**Fig. 3:**
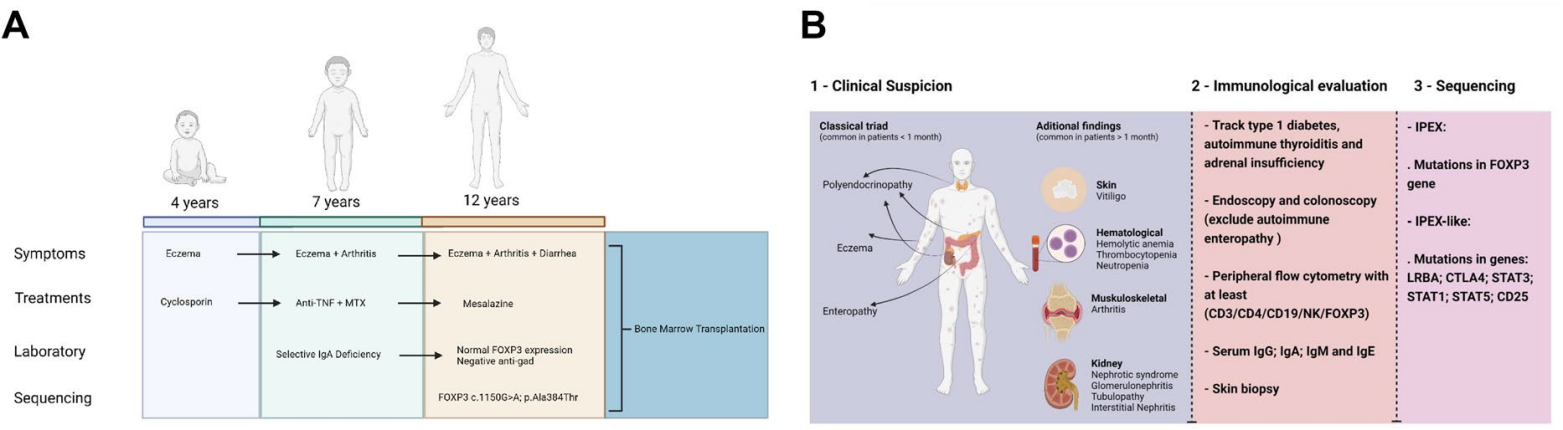
**A** Timeline of disease evolution, treatments and genetic sequencing. **B** Practical flowchart to diagnosis and red flags for IPEX/IPEX-like syndromes

Immunological aspects of IPEX also deserve a special overview. Selective immunoglobulin A deficiency (SIgA-D) is a common humoral immunodeficiency worldwide found to occur in 1:965 individuals in Brazil, and usually it is the first manifestation of many other inborn errors of immunity [13]. SIgA-D is not common in IPEX syndrome and usually low levels of all immunoglobulins is only observed in patients with severe enteropathy which was absent in our case in the moment SIgA-D was diagnosed [2]. Therefore, we suggest that patients with SIgA-D associated with several immunedysregulatory phenomena, even in the absence of autoimmune endocrine diseases, should be screened for FOXP3 mutations (Fig. 3B). Different patterns of peripheral FOXP3 expression have been reported in IPEX syndrome and differently from what is expected, normal to high expression can also be observed [5, 9, 14]. For instance, patients with IPEX syndrome that lack the genetic finding (a.k.a. IPEX-like syndrome) and have low expression of FOXP3 can have a broad spectrum of genetic basis, which demonstrates the genetic variability associated to the same phenotype [9].

The standard of care for IPEX patients is hematological stem cell transplantation. Immunosuppressive therapies in IPEX patients that not underwent HSCT demonstrates that the association of steroids with both calcineurin inhibitors and non-calcineurin inhibitors can successfully control autoimmunity. Additionally, the use of monoclonal antibodies, such as anti-TNF, to the immunosuppressive therapy seems to be effective in controlling autoimmunity but studies that are more robust are needed. However, data consensus regarding the specific moment that HSCT should be indicated is not available and the same occurs for the ideal drug to immune modulate mild inflammatory phenotypes or those not transplanted [9, 11].

The first description of A384T related to IPEX dates from 2001 in a large family with many premature deaths. At that time, the authors found three patients alive in the same family harboring the mutation A384T without more clinical details [15, 16]. Although the residue threonine at the position 384 is similar to the most common amino acid found in this residue, animal models demonstrated that this mutation leads to IPEX syndrome and therefore influences the homeostasis of T-cell activation. Later, genotype–phenotype correlations found that mutations localized in the forkhead domain of the gene, as here, reported, were more significantly associated to alopecia areata as found in our patient. However, other phenotypes not observed in our patient were also found to be statistically significant in the same domain, such as anemia. Curiously mutations along the poly A seems to be more related to immunedysregulatory manifestations, whether the mutation herein reported is located in the distal part of the gene and is clinically represented of many immunedysregulatory findings [2].

## Conclusion

IPEX syndrome is a rare but often fatal multi systemic autoimmune disease that presents with a heterogeneity of phenotypes. Therefore, it is imperative to be considered as a diagnosis in patients affected by multiple autoimmune/inflammatory phenomena even if those manifestations trespass the classic clinical description of the syndrome and regardless of their onset. Special attention shall be paid when screening individuals with peripheral FOXP3 expression since normal levels can have a genetic background and many other genes can be implicated in low expression of FOXP3. The widespread use of large panels/exome sequencing has guaranteed the final correct diagnosis and has prompted arrangement for adequate interventions and treatments resulting in patient’s improved quality of life and survival.

## Supplementary Information

**Additional file 1.**1 - FOXP3 expression in the patient compared to the mother and the healthy control. The flow figures from FMO to patient, mother and healthy donor cytometry flow acquisitions.

## Data Availability

The datasets used and/or analyzed during the current study are available from the corresponding author on reasonable request. Figures were generated with Biorender.

## Abbreviations

IPEX: Immune dysregulation, polyendocrinopathy, enteropathy, X-linked
FOXP3: Forkhead Box 3
DNA: Deoxydribonucleic acid
CD: Cluster differentiation
S-IgAD: Selective immunoglobulin A deficiency
