# Supplementary material for: Underlying IPEX syndrome in a patient with idiopathic juvenile arthritis and vitiligo
Source: Allergy Asthma Clin Immunol. 2022 Dec 12;18:105. doi: 10.1186/s13223-022-00740-9 (PMC9743487; doi:10.1186/s13223-022-00740-9)

Supplementary Files

1 – Graphical demonstration of peripheral expression of FOXP3 in the patient, the mother and in the healthy control

2 – Flow cytometry FMO files and plots acquision of FOXP3 in the patient and in the mother

A – FMO FOXP3 acquisition healthy control


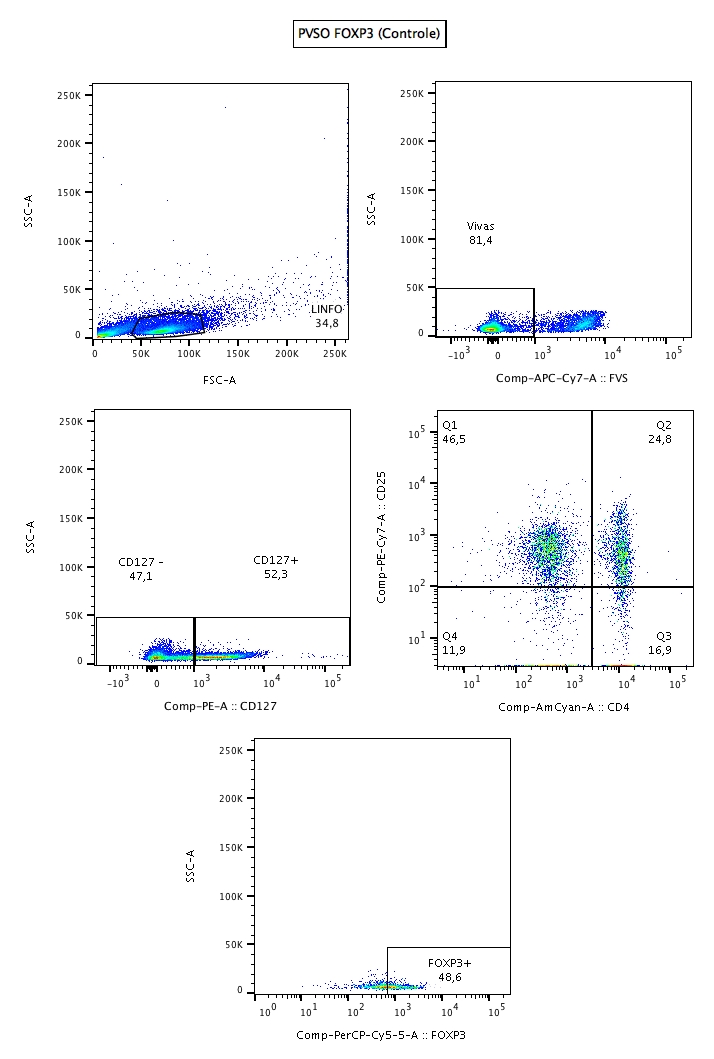


B - A – FMO FOXP3 acquisition proband


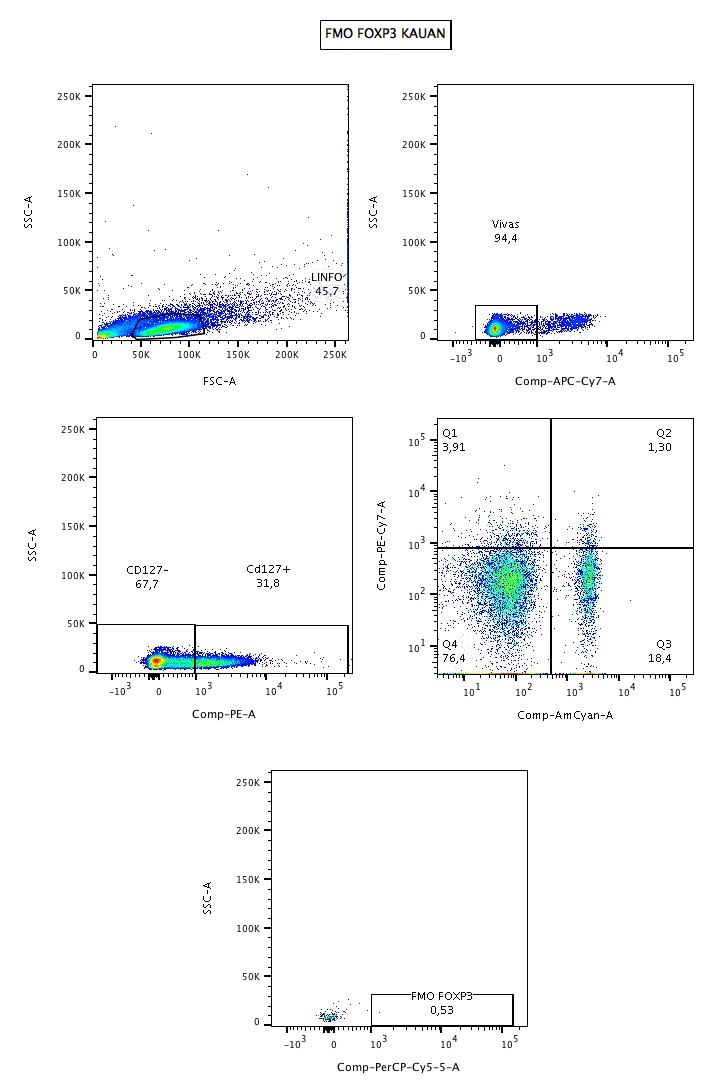


B - A – FMO FOXP3 acquisition mother


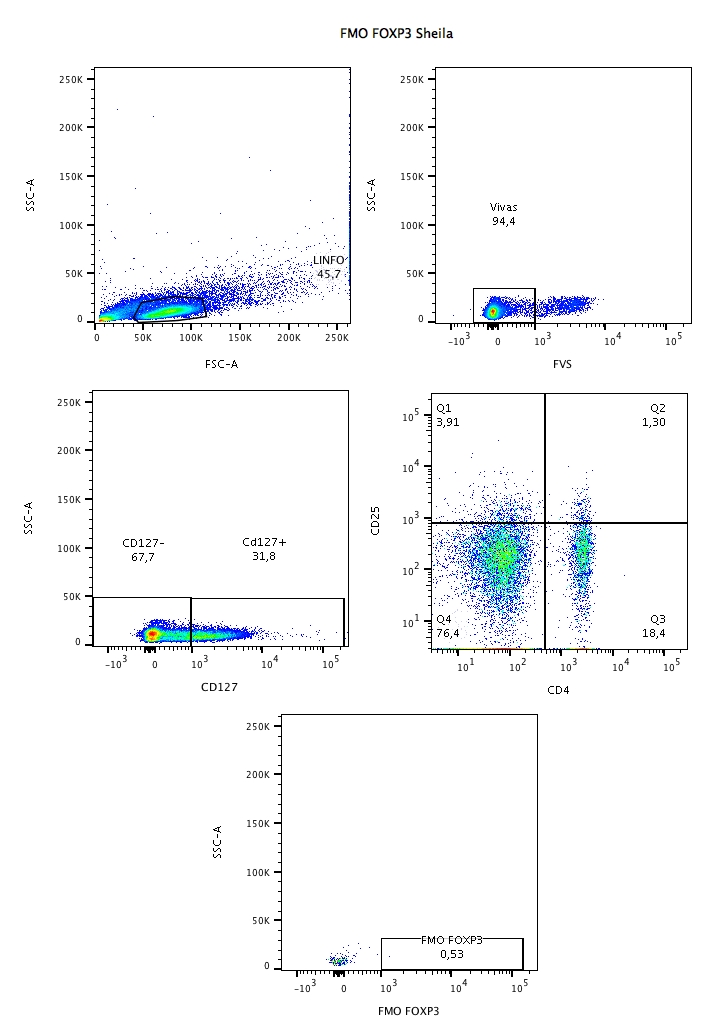

Supplement: Supplementary file 1 — Additional file 1.1 - FOXP3 expression in the patient compared to the mother and the healthy control. The flow figures from FMO to patient, mother and healthy donor cytometry flow acquisitions. [file 13223_2022_740_MOESM1_ESM.docx]
